# The USA lags behind other agricultural nations in banning harmful pesticides

**DOI:** 10.1186/s12940-019-0488-0

**Published:** 2019-06-07

**Authors:** Nathan Donley

**Affiliations:** Environmental Health Program, Center for Biological Diversity, P.O. Box 11374, Portland, OR 97211 USA

**Keywords:** Pesticide, Regulation, Agriculture, Environmental Protection Agency

## Abstract

**Background:**

The United States of America (USA), European Union (EU), Brazil and China are four of the largest agricultural producers and users of agricultural pesticides in the world. Comparing the inclination and ability of different regulatory agencies to ban or eliminate pesticides that have the most potential for harm to humans and the environment can provide a glimpse into the effectiveness of each nation’s pesticide regulatory laws and oversight.

**Methods:**

The approval status of more than 500 agricultural pesticides was identified in the USA, EU, Brazil and China and compared between nations. The amount of pesticides that were used in the USA and banned in these other nations was compiled and linear regression was used to identify trends in use.

**Results:**

There are 72, 17, and 11 pesticides approved for outdoor agricultural applications in the USA that are banned or in the process of complete phase out in the EU, Brazil, and China, respectively. Of the pesticides used in USA agriculture in 2016, 322 million pounds were of pesticides banned in the EU, 26 million pounds were of pesticides banned in Brazil and 40 million pounds were of pesticides banned in China. Pesticides banned in the EU account for more than a quarter of all agricultural pesticide use in the USA. The majority of pesticides banned in at least two of these three nations have not appreciably decreased in the USA over the last 25 years and almost all have stayed constant or increased over the last 10 years.

**Conclusions:**

Many pesticides still widely used in the USA, at the level of tens to hundreds of millions of pounds annually, have been banned or are being phased out in the EU, China and Brazil. Of the pesticides banned in at least two of these nations, many have been implicated in acute pesticide poisonings in the USA and some are further restricted by individual states. The United States Environmental Protection Agency (US EPA) has all but abandoned its use of non-voluntary cancellations in recent years, making pesticide cancellation in the USA largely an exercise that requires consent by the regulated industry.

**Electronic supplementary material:**

The online version of this article (10.1186/s12940-019-0488-0) contains supplementary material, which is available to authorized users.

## Background

Four of the largest agricultural producers in the world are the USA, EU, China, and Brazil – together accounting for more than half of all global agricultural production value [1]. In addition, these four nations have the highest export values of any other agricultural producers in the world and, therefore, have an enormous economic interest in maintaining high production [1].

Many agricultural practices can be harmful to humans and surrounding ecosystems and their potential benefits must be balanced against these harms [2]. One widely adopted agricultural practice that is known to have harmful impacts to humans and the environment is the use of pesticides. While many pesticides are efficacious against agricultural pests and widely used to prevent crop damage, the harms to non-target species and humans can be widespread and severe [3, 4]. In addition to being the world’s largest agricultural producers and exporters, the EU, Brazil, USA, and China are some of the world’s largest pesticide users – each using 827 million, 831 million, 1.2 billion, and 3.9 billion pounds of pesticides in 2016, respectively [5–7].

The USA, EU, China, and Brazil each have separate and distinct pesticide regulatory systems designed to protect, to varying degrees, humans and the environment. The EU, consisting of 28 member states, currently has the most comprehensive and protective pesticide regulations of any major agricultural producer. The European Commission oversees pesticide approval, restriction and cancellation in the EU in accordance with Regulations 1107/2009 and 396/2005, which are designed to “…ensure that industry demonstrates that substances or products produced or placed on the market do not have any harmful effect on human or animal health or any unacceptable effects on the environment” and place the burden of proof on the pesticide industry to demonstrate that its product can be used in a way that does not result in harm to humans or the surrounding environment [8, 9]. The EU prohibits the approval and continued use of pesticides that the governing body has recognized as mutagens, carcinogens, reproductive toxicants or endocrine disruptors unless exposure to humans is considered negligible [8].

In the USA, pesticide regulation is largely overseen by the US EPA, which regulates and enforces pesticide actions under the Federal Food, Drug, and Cosmetic Act (FFDCA) and the Federal Insecticide, Fungicide, and Rodenticide Act (FIFRA) [10, 11]. Unlike the safety threshold afforded by the EU, the pesticide industry only has to demonstrate that its products “will not generally cause unreasonable adverse effects on the environment,” which is partially defined as “any unreasonable risk to man or the environment, taking into account the economic, social, and environmental costs and benefits of the use of any pesticide…” [11]. The FFDCA was amended in 1996 to strengthen the safety threshold in setting food residue tolerances to a “reasonable certainty of no harm” for pesticide exposure to humans through food, water and home uses [12]. However, harm to plants, animals, the broader environment, and harm to humans from occupational exposures remains solely a cost-benefit analysis.

Historically, pesticide regulation in China has suffered from scattered data, complex laws and lack of transparency regarding rule implementation and compliance [13]. Recently, China has passed modest regulations updating certain aspects of pesticide use in the country, including establishing licensing requirements for sellers of pesticides, record keeping requirements for users, and committees in charge of evaluating pesticide safety [14]. One notable area where China has progressed in recent years is with banning or phasing out highly hazardous pesticides. As of 2014 the Chinese Ministry of Agriculture (MOA), the lead pesticide regulatory agency which upholds the newly revised Pesticide Management Law, had banned or was in the process of phasing out 50 pesticides and in the process of restricting another 30 [15]. More recent regulations have resulted in the announced phase out of an additional 12 pesticides by 2022 [16].

Brazil’s pesticide regulations are overseen by three governmental agencies, the Brazilian MOA, Brazilian Health Regulatory Agency (ANVISA) and Ministry of the Environment (MOE) [17]. Under Brazil’s 1989 pesticide law No. 7802, the country incorporated a more protective “hazard assessment” by which it can ban carcinogenic, teratogenic, mutagenic and hormone disrupting pesticides [18]. However multiple factors have severely limited the effectiveness of human and environmental health safeguards in Brazil, including: 1) barriers to how often pesticides can be reevaluated, 2) the Brazilian MOA’s aggressive protection of the agrochemical industry, and 3) massive budget and personnel shortfalls [18, 19]. Despite this, ANVISA and the Brazilian MOE have been effective in getting some hazardous pesticides banned in the country [20].

While regulatory agencies have many options to increase the safeguards for any given pesticide, including limiting what crops the pesticide can be used on, requiring safety equipment to be worn by applicators, requiring setbacks from sensitive habitats, and requiring management practices to minimize off-target movement, the most effective and reliable option is to ban a pesticide entirely if the potential for dangerous exposure cannot be feasibly mitigated. As such, one measure of the effectiveness of a regulatory agency is how it compares to its peer agencies in banning or eliminating pesticides that are most dangerous and have the most potential for harm to humans and the environment.

A recent decision by former US EPA Administrator Scott Pruitt that reversed a planned ban on the pesticide chlorpyrifos, as well as the increasing influence of the agrochemical industry in the operations of US EPA, has called into question the effectiveness and robustness of pesticide regulation in the USA [21, 22]. Here, I identified pesticides that are approved in outdoor agricultural applications in the USA and compared to those in the EU, China and Brazil. Many pesticides are still widely used in the USA that have been banned in these other nations and the majority of pesticides banned in at least two of them have not appreciably decreased in use in the USA over the last 25 years. The number of US EPA-initiated, non-voluntary cancellations in the USA has decreased substantially in recent years making pesticide prohibitions largely a result of voluntary cancellations by industry. Finally, I discuss potential influencing factors, as well as the negative implications for human health and the environment in the USA.

## Methods

### Pesticide approval status

A list of more than 500 pesticide active ingredients that have been used in agriculture in the USA, EU, Brazil and China was compiled for use in comparing the approval status between nations (Additional file 1 and Additional file 2). Pesticide Action Network (PAN) International maintains a list of pesticides that are banned in various countries [23]. While the PAN database is comprehensive and updated regularly, its drawbacks for this analysis are: 1) it is incomplete with respect to pesticide status in the USA; 2) the most recent source for pesticide status in China is from 2014; 3) it does not separate voluntary pesticide cancellation from non-voluntary cancellation in the USA and EU; and 4) the list does not separate pesticides used in agriculture from other uses. Therefore, this analysis was done independently of the PAN International list; however, many of the same sources were used.

In order to compare pesticide bans between different countries, it is imperative to define a pesticide “ban.” China and Brazil both issue bans to forbid the use of certain pesticides in agriculture. Therefore, determining whether these countries have banned a pesticide is a very straightforward process. The USA and EU do not technically ban pesticides; they simply revoke the approval of a pesticide – which acts as a de facto ban because an unapproved pesticide cannot be used in those jurisdictions. While bans in China and Brazil are generally related to pesticide safety, there are multiple reasons a pesticide approval can be revoked or cancelled in the EU and USA. These include safety concerns, failure of the registrant to pay fees or submit required studies, or the pesticide registrant has voluntarily requested registration be cancelled for economic or other reasons. For this study, a pesticide in the USA and EU was considered “banned” if a decision was made by the regulating agency to unilaterally prohibit a pesticide from entering the market, cancel its approval, or notify the Rotterdam Convention that the pesticide was banned. A pesticide was considered “not approved” if a pesticide registrant voluntarily withdrew its application, voluntarily requested that registration be cancelled, the registration expired or the pesticide has never been approved. This was done to separate regulatory actions that were taken to protect human and environmental health (banned) from those that were taken for economic or other reasons (not approved).

Using the sources identified in Additional file 3: Tables S6-S9, each agricultural pesticide was designated as “approved,” “not approved,” “banned” or “in the process of phase out” in the USA, EU, Brazil, and China (see also Additional file 1). If a pesticide’s status could not be identified it was designated as “not in database/unknown.”

### Pesticide use data and trends in the USA

The United States Geological Survey (USGS) National Water-Quality Assessment Project maintains an online resource of annual pesticide use estimates for all pesticides used in USA agriculture from 1992 forward [7]. A description of how these estimates are generated can be found in Thelin and Stone, 2013 [24]. To obtain total annual pesticide use, I downloaded 2016 preliminary pesticide use estimates and separated them by pesticide in Additional file 4. Use data in all states and counties were totaled for each pesticide and converted from kilograms to pounds. Upper-end estimates (E-Pest High) were totaled for all pesticides that are used in the USA and banned or being phased out in at least one other nation (Additional file 5).

For trends in use over time, pesticide use data were obtained as described above from 1992 to 2016 for the following pesticides that are approved in the USA but banned or being phased out in at least two of the three compared nations: 2,4-DB, bensulide, chloropicrin, dichlobenil, dicrotophos, EPTC, norflurazon, oxytetracycline, paraquat, phorate, streptomycin, terbufos, and tribufos. Data were plotted over the 25-year period and over the most recent 10 years. To determine if pesticide use significantly changed over time, a linear regression was conducted for each pesticide over the 25- and 10-year period in SPSS for Windows version 25.0. To ensure normal distribution and homoscedasticity of residuals, pesticide use numbers from some pesticide datasets were natural logarithm-transformed prior to statistical analysis. Data were normally distributed for all datasets as measured by Shapiro-Wilk (*p* > 0.05), with the sole exception of oxytetracycline use over a 25-year period (*p* = 0.001).

## Results

**Fig. 1:**
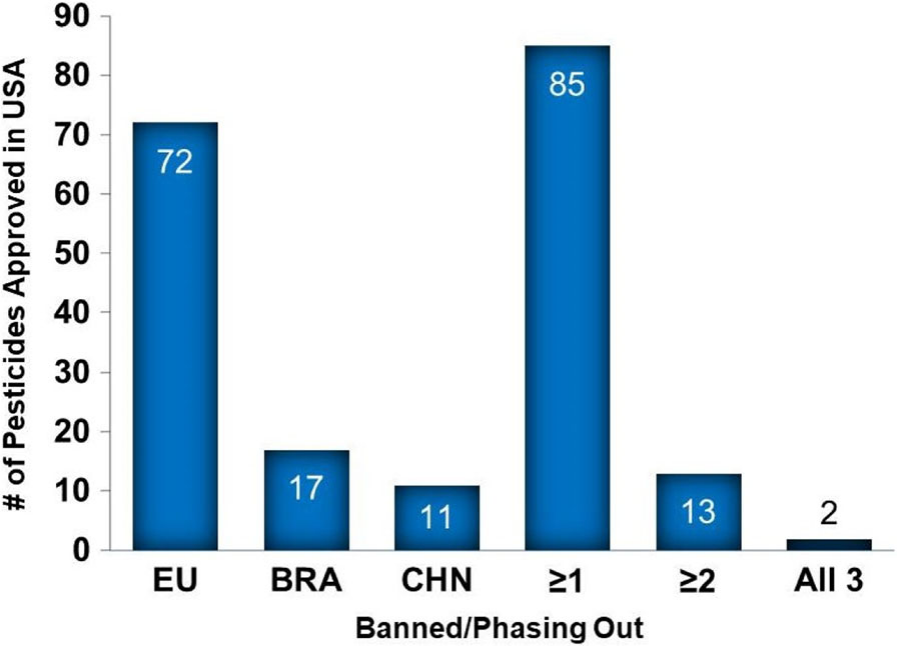
The number of pesticides approved for outdoor agricultural use in the USA that are banned or being phased out in the European Union (EU), Brazil (BRA), China (CHN), at least one of the three (≥1), at least two of the three (≥2) or all 3

There have been over 500 active pesticide ingredients used in agricultural applications in the USA since 1970, the year the US EPA was formed (Additional file 2: Table S5). A comparison of the approval status of each of these pesticides indicates that 72, 17, and 11 pesticides that are approved for outdoor agricultural applications in the USA are banned or in the process of complete phase out in the EU, Brazil, and China, respectively (Fig. 1 and Additional file 3: Tables S11-S13). In addition, 85, 13, and two pesticides were identified as being approved in the USA and banned or in the process of phase out in at least one of the three, two of the three, or all three agricultural nations, respectively (Fig. 1 and Additional file 3: Tables S14-S16). This compares with two, three, and two pesticides that have been banned in USA agriculture that are approved for use in the EU, Brazil and/or China, respectively (Additional file 3: Table S19). Of the 85 pesticides approved in the USA and banned in at least one of the other nations, most are herbicides (58%) followed by insecticides (20%), fungicides/nematicides/bactericides (16%) and those having both insecticide/fungicide activity (6%) (Additional file 3: Table S18).

Of the 1.2 billion pounds of pesticides used in USA agriculture in 2016, roughly 322 million pounds were of pesticides banned in the EU, 40 million were of pesticides banned in China and nearly 26 million were of pesticides banned in Brazil (Table 1 and Additional file 5: Tables S131-S133). More than 10% of total pesticide use in the USA in 2016 was from pesticide ingredients either banned, not approved or of unknown status in all three agricultural nations (Table 1 and Additional file 5: Table S137).

**Table 1:** Total Agricultural Pesticides Used in the USA and Banned in the EU, Brazil or China

	Lbs. Pesticides Used in USA Agriculture	% of Total
Total	1,200,587,514	100
Banned in EU	322,597,233	26.9
Banned in CHN	40,014,277	3.3
Banned in BRA	25,843,457	2.2
Banned in at Least 1	327,817,174	27.3
Banned in at Least 2	45,960,605	3.8
Banned in All 3	14,677,188	1.2
Banned, Not Approved or Unknown in All 3	133,711,048	11.1

The total pounds (lbs.) of pesticides used in agricultural applications in the USA in 2016 categorized by where they are banned or being phased out. The last row indicates pesticides that are banned, not approved or of unknown status in the European Union (EU), China (CHN) and Brazil (BRA)

**Fig. 2:**
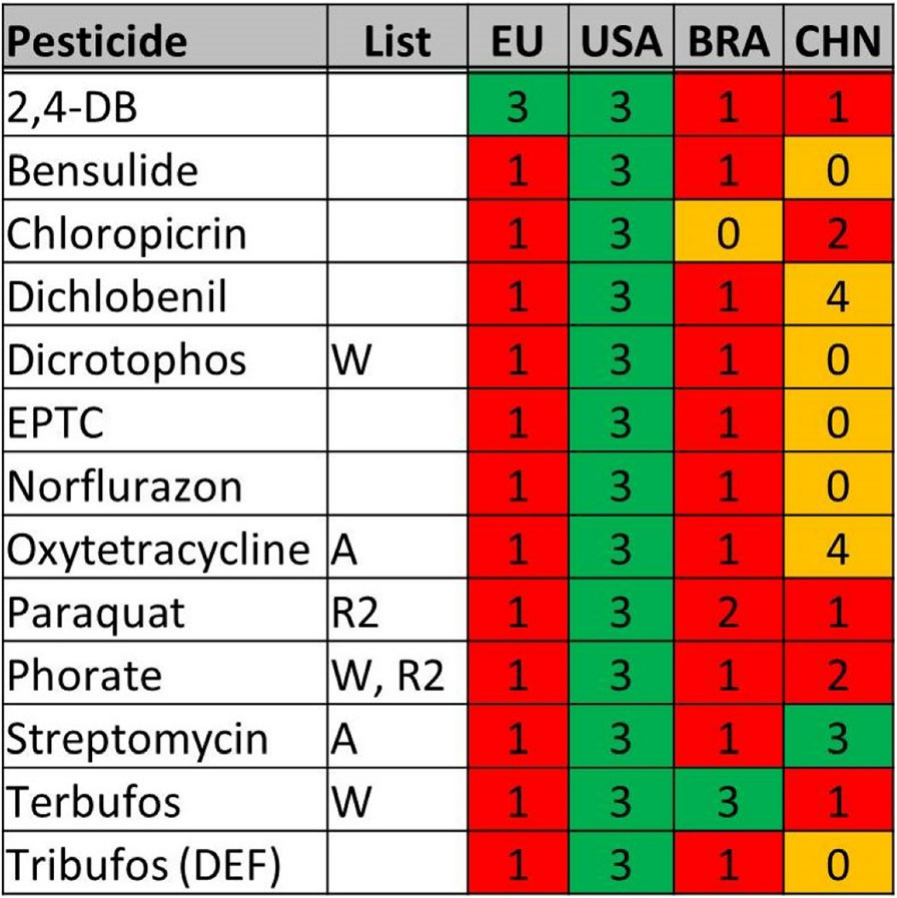
Pesticides Used in the USA and Banned in at Least Two of Three Other Agricultural Nations. The first column gives the common pesticide name. The second column indicates whether the pesticide is on an international list of concern (W=World Health Organization (WHO) “extremely” or “highly” hazardous pesticide [79]; R2 = Rotterdam Convention Annex III list, Recommended [73]; A = WHO “critically” or “highly” important antibiotics [53]). Columns 3–6 indicate the pesticide status in the European Union (EU), the United States of America (USA), China (CHN) or Brazil (BRA). 1 = Banned; 2 = In process of complete phase out; 3 = Approved; 4 = Not approved/voluntarily withdrawn; 0 = Not in database/unknown. Red = banned/phasing out; Green = approved; Orange = Not approved/unknown

Over 45 million pounds of agricultural pesticide use in the USA comes from the 13 pesticides that are banned or in the process of phase out in at least two of the three other agricultural nations (Table 1 and Additional file 5: Table S135). Paraquat and phorate are the only two pesticides that are banned or being phased out in all three places, however 10 of the 13 are either banned, being phased out, not approved or of unknown status in all three (Fig. 2).

**Fig. 3:**
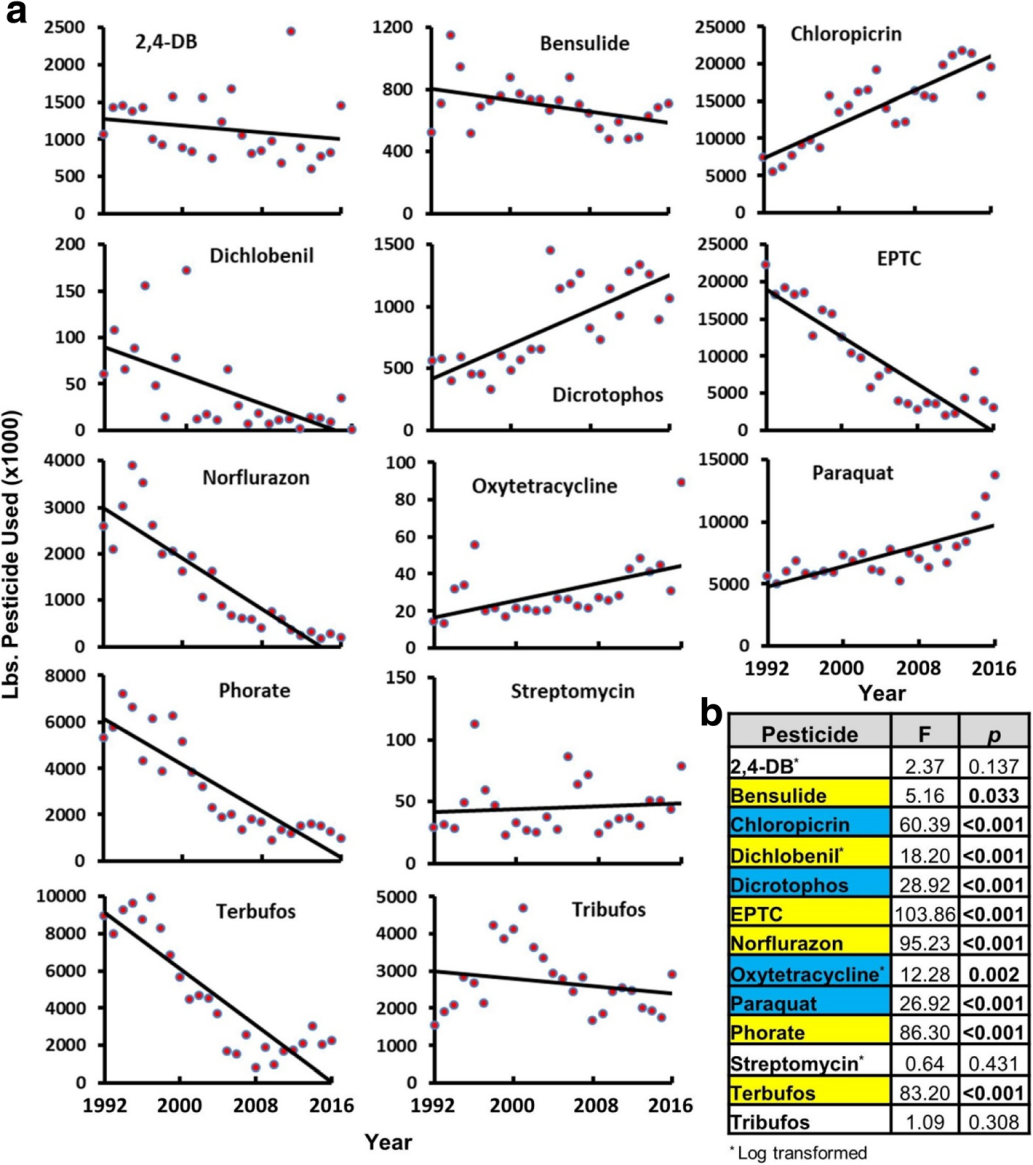
Trends in Use of Pesticides in the USA that are Banned in at Least Two of Three Other Agricultural Nations. **a)** Total pesticide use in the USA in pounds (lbs.) was plotted for each year between 1992 and 2016 for each of 13 pesticides that have been banned or are being phased out in at least two of the following places: the EU, China and Brazil. Each graph contains a linear trend line. **b)** Results of linear regression analyses that were conducted for each pesticide over the 25-year period. Data were log-transformed where indicated and the degrees of freedom (df) for each pesticide dataset equals 24 with the exception of dichlobenil (df = 23; the zero value for 2016 was removed before log transformation). Bold *p*-values were statistically significant (*p* < 0.05). Pesticides highlighted in yellow had a significant downward trend, pesticides highlighted in blue had a significant upward trend and those that were not highlighted had no significant change over time

From 1992 to 2016, the trends in use of the 13 pesticides that are banned in at least two other places and used in the USA varied by pesticide (Fig. 3). Bensulide, dichlobenil, EPTC, norflurazon, phorate, and terbufos all significantly decreased over this 25 year period, with five of the six showing a very steep decrease in use. Four of the pesticides – chloropicrin, dicrotophos, oxytetracycline and paraquat – significantly increased over this time period indicating a greater demand for use concomitant with no significant additional restrictions. Use of 2,4-DB, streptomycin and tribufos did not significantly change over this time period. Many of the pesticides that decreased in use over the last 25 years showed a marked plateau in recent years (Fig. 3). Over the more recent 10 years (2007–2016), only one pesticide, norflurazon, significantly decreased in use, while oxytetracycline and paraquat had significantly increased (Additional file 6).

**Fig. 4:**
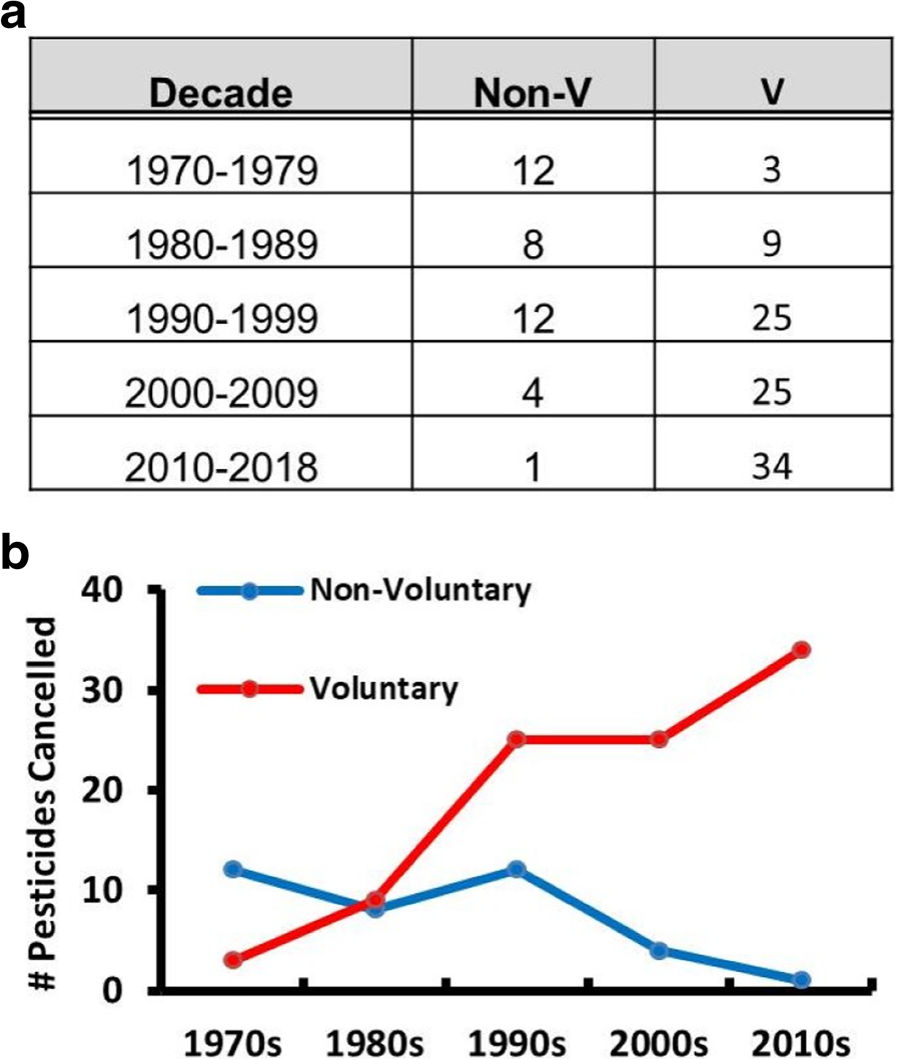
Number of Agricultural Pesticides Cancelled in the USA by Decade from 1970 to the Present. **a)** Table presenting the number of agricultural pesticides that were non-voluntarily (Non-V) or voluntarily (V) cancelled in the USA by decade. **b)** Graphic representation of table in **a.**

Of the 508 pesticide active ingredients that have been used in agriculture in the USA since 1970, 134 have been cancelled (Additional file 3: Table S9). Of those 134, 97 have been voluntarily cancelled by pesticide registrants or had a time-limited approval that expired. That leaves 37 pesticides where the US EPA took unilateral action to prohibit an agricultural pesticide from entering the market or cancel its approval. Many of these 37 are highly persistent, dangerous pollutants that have triggered massive public outcry in the USA and throughout the world, such as aldrin, DDT, dieldrin, chlordane, carbofuran and toxaphene. Broken down by decade, the bulk of these decisions came before the year 2000, with only five agricultural pesticides being non-voluntarily cancelled in the last 18 years (Fig. 4). Cancellations voluntarily requested by the pesticide registrant have greatly increased in the last 40 years and currently account for nearly all agricultural pesticide cancellations in the USA.

## Discussion

As four of the largest agricultural producers, the EU, China, Brazil and USA have an outsized role in the generation of agricultural commodities used throughout the world. Each nation has its own regulations and rules regarding the use of pesticides in agriculture. This study sought to identify the pesticides these different regulatory systems have deemed too dangerous to use at any level and compare between nations. In addition to being major agricultural producers, the EU, Brazil and China are also some of the largest users of agricultural pesticides in the world – making them ideal for comparison with the USA [5–7].

The main focus of this study was on the 13 pesticides that are approved in the USA but banned in at least two other large agricultural nations (Fig. 2). There are a couple of reasons that could explain why these pesticides remain in use in the USA, and in some cases are even increasing, while having been banned by multiple other peer regulatory agencies. One possibility is that the USA has unique pest problems that necessitate the use of these harmful pesticides in agriculture. 2,4-DB, bensulide, dichlobenil, EPTC, norflurazon, and paraquat are herbicides that are used in the USA to kill problem weeds in crops that are also grown in China, Europe and Brazil, like soybeans, corn, fruits and vegetables, nut trees, cotton, peanuts and wheat. Problem weeds are not unique to the USA and the US EPA pesticide labels for each of these herbicides list efficacy against weeds that are also a common agricultural nuisance in places where the herbicides are banned [25–28]. Tribufos is not used to kill pests in the USA but as a defoliant to increase the harvest efficiency of cotton, a crop that is widely grown in Brazil and, to a lesser extent, Europe [29]. Dicrotophos, also used solely on cotton in the USA, is labelled as being effective against cotton pests that exist in Brazil and Europe [30, 31]. Terbufos is used mainly on corn in the USA and its US EPA label claims efficacy against multiple agricultural pests that exist in Chinese and European corn crops [32, 33]. Phorate and chloropicrin are used on a wide variety of crops in the USA, mainly commodity crops for the former, and fruits and vegetables for the latter; both have broad-spectrum pest control and efficacy against common agricultural pests in Brazil, China and Europe. Oxytetracycline and streptomycin are approved in the USA to combat fire blight and bacterial spot in certain fruit trees, diseases which also have spread in Europe and Brazil [34, 35]. This indicates that these pesticides could have utility in agriculture in these countries were they not found to be too harmful for human and environmental health.

Since the US EPA will often place use restrictions on pesticide labels as a way of mitigating harm to humans and the environment, another possibility could be that the USA is effectively safeguarding against harm without having to resort to a complete ban. However, five of the thirteen pesticides used in the USA that are banned in at least two of three agricultural nations are neurotoxic pesticides of the organophosphate (OP) class (bensulide, dicrotophos, phorate, terbufos, and tribufos). Over 2000 incidents involving OPs were reported to poison control centers across the USA each year from 2012 to 2016 [36–40]. The vast majority of these poisonings were accidental in nature and range in severity from minor to, in some cases, death. Data from the National Institute for Occupational Safety and Health indicate that between 1998 and 2011, 43% of insecticide related illnesses in the USA involved cholinesterase inhibitors like OPs [41]. Paraquat, one of the most acutely lethal pesticides still in use today, is implicated in around 100 poisoning incidents in the USA each year, resulting in at least one death per year since 2012. Of reported poisoning events in the USA involving paraquat as a single agent from 2012 to 2016, anywhere from 84 to 94% were accidental (unintentional) in nature [36–40]. The US EPA’s human Incident Data System identified 27 deaths, 22 high severity incidents and 181 moderate severity incidents involving paraquat from 1990 to 2014 [42]. From 2000 to 2015, agricultural usage of chloropicrin was implicated in over 1000 pesticide-related illnesses in California alone [43]. Acute pesticide poisonings in agriculture also remain severely underreported due to language barriers, fear of deportation or job loss and the economic disadvantage of those most highly exposed, so these numbers are likely under-representative of the true impact [44]. Thus, while the US EPA can place restrictions on pesticide labels, if people have ready access to extremely toxic pesticides, accidents and misuses are inevitable and can lead to severe consequences for those involved.

In addition to numerous incidents of acute poisonings, multiple states have determined that current US EPA regulations are not protective enough for some of these pesticides and have opted to place greater restrictions on use than the US EPA requires. California – the largest agricultural producing state in the USA by value – has imposed greater restrictions on chloropicrin, EPTC and norflurazon, including larger buffer zones, reduced acreage that can be treated, additional protective equipment and mitigations to prevent groundwater contamination [45–48]. The state of New York has banned phorate in certain counties and aerial application of the pesticide in the entire state [49]. Certain counties in Washington state have prohibited aerial spraying of paraquat [50–52].

Furthermore, two of the 13 pesticides, streptomycin and oxytetracycline, are antibiotics that are recognized as “critically” and “highly” important for human medicine by the World Health Organization (WHO), respectively [53]. Overuse and abuse of medicines like these can accelerate the development of antibiotic resistant bacteria, which the Centers for Disease Control and Prevention (CDC) estimate infect at least two million people and result in the deaths of 23,000 people annually [54]. Non-human use of antibiotics in agriculture is known to be one way that antibiotic resistant bacteria can develop and spread to humans and, while most antibiotics in agriculture are used on animals that are kept in confined spaces, the use of antibiotics directly on crops can result in a considerable area of land being exposed on a semi-regular basis [55]. Roughly 80,000 pounds each of streptomycin and oxytetracycline were used on plants in the USA in 2016 (Additional file 4: Tables S92, S116). With the 2018 US EPA approval of oxytetracycline on citrus crops, use of this antibiotic is expected to increase to more than 388,000 pounds per year – 130,000 pounds more than all tetracyclines used annually in human medicine in the USA [56, 57]. A similar impending increase in streptomycin use, which the US EPA proposed at the end of 2018, indicates that the use of these antibiotics will continue to increase in future years, despite the risk of resistance genes developing in human pathogens [55, 58]. Altogether, it appears that the US EPA has not taken sufficient action to meaningfully reduce use of, and risks from, pesticides that are banned in multiple other nations by simply placing mitigation measures on the pesticide label.

During this analysis it became clear that the USA utilizes voluntary (industry-initiated) cancellation as the primary method of prohibiting pesticides, which is different than the non-voluntary (regulator-initiated) cancellations/bans that predominate in the EU, Brazil and China. In fact, it is now almost exclusively the sole method the US EPA uses to cancel agricultural pesticides (Fig. 4). There are likely several reasons for this. FIFRA was amended in 1988 to implement annual maintenance fees on pesticide registrations and increase data requirements [59]. It was amended again in 2004 with the Pesticide Registration Improvement Act that increased registration fees in exchange for accelerated registration decisions [60]. The passage of these two amendments correlates with two big jumps in voluntary cancellations over the last 50 years (Fig. 4). This would be expected, as the more it costs to comply with registration requirements the more likely it is that poor-selling pesticides or those that are no longer effective due to pest resistance issues will not justify the cost of maintaining registration in the USA. Furthermore, as patent protection on pesticides and exclusive use periods for data protection expire, the registration holder may be more likely to voluntarily cancel the registration – particularly if generic products have flooded the market or if a company wants to shift its resources to a newer active ingredient that has those protections still in place [61]. And in a time of intense consolidation in the pesticide industry, lower performing, redundant and competing products are more likely to be voluntarily cancelled, indicating that voluntary cancellations due to economic reasons may be on the rise in the near future. Therefore, many of these voluntary cancellations are likely business decisions made by the registrants and can be influenced by any number of economic factors.

On the other hand, there are also instances when voluntary cancellations are used as a negotiating tool by the US EPA or would not have been requested without some amount of regulatory pressure. For instance, mevinphos was voluntarily cancelled in the USA by the registrant once the US EPA made it clear that it intended to suspend the pesticide due to human health concerns [62]. With aldicarb, the manufacturer agreed to an extended voluntary phase-out in exchange for the US EPA not initiating cancellation proceedings [63]. Additionally, of the 20 agricultural OP pesticides that have been voluntarily cancelled in the USA, 10 were cancelled after the Food Quality Protection Act (FQPA) amendment to FIFRA began to be implemented in the early 2000s (Additional file 3: Table S20) [12]. Nine of those 10 were used on food crops and the stricter safety requirements of the FQPA regarding food exposures likely played a role in the voluntary removal of those pesticide ingredients, as it is believed to be a contributing factor in decreased OP use over the last 20 years [64].

Overall, voluntary cancellations in the USA appear to have played a role in facilitating the removal of some potentially hazardous pesticides. But while voluntary cancellations have one benefit—that being a certainty that the cancellation will not be challenged in court by the pesticide registrant—there are notable downsides to using this as the primary method of cancelling pesticides. The major one being that it requires at least some desire on the part of the pesticide registrant. All 10 agricultural OP pesticides that were voluntarily cancelled in the USA after 2002 had already steeply decreased in use before they were cancelled, suggesting that the economic benefits of their continued registration were not as favorable to the pesticide industry (Additional file 3: Table S20) [7]. This contrasts with other OPs that have not been cancelled in the USA and whose uses have remained high and relatively stable over time, like acephate, bensulide, chlorpyrifos, dimethoate and malathion [7]. It’s likely that the reason some OPs have been voluntarily cancelled while others remain approved in the USA reflects registrants’ willingness or unwillingness to voluntarily cancel or negotiate a voluntary cancellation with the US EPA.

Not only do voluntary cancellations ultimately bias towards pesticides that are easier to cancel because they are less economically valuable to pesticide makers, but they can lead to a significantly longer phase out period. For example, instead of initiating a notice of intent to cancel aldicarb for posing unacceptable risks to infants and young children in 2010, the US EPA entered into a signed agreement with the registrant to voluntarily cancel the pesticide [63]. This agreement allowed the registrant to continue manufacturing the pesticide for four years with a complete phase out achieved in another four years. This eight-year phase out contrasts sharply with the typical one-year phase out for most cancelled pesticides [65].

Under FIFRA, US EPA-initiated cancellation is a time-consuming process, requiring considerable agency resources and multiple steps designed to ensure, above all, that the agricultural sector will not experience undue hardship. After the US EPA decides to initiate cancellation, it must notify the US Department of Agriculture and the FIFRA Scientific Advisory Panel of its decision and respond to any concerns they may have. Following that, the registrant can request a hearing with an administrative law judge and that decision can be appealed to an appeals board where the US EPA “… is required by FIFRA to consider restricting the use of the pesticide as an alternative to cancellation while explaining the reasons for the restrictions and taking into account the effect of such final action on production and prices of agricultural commodities, retail food prices, and otherwise on the agricultural economy” [66]. During the appeal process, the pesticide approval remains in place and it can continue to be used.

Despite all of this, the US EPA has occasionally been successful using non-voluntary cancellation to achieve bans on certain pesticides – even in recent years. After finding that carbofuran resulted in unacceptable harms to humans through diet in 2009, the agency was ultimately successful in forcibly cancelling the pesticide – even after the registrant challenged the decision all the way to the US Supreme Court [67, 68]. The agency also succeeded in non-voluntarily cancelling flubendiamide in 2016 after the registrant reneged on its commitment to voluntarily cancel the pesticide if the US EPA identified significant harms after further review [69]. However, the US EPA has also been unsuccessful in its efforts to cancel a pesticide when industry does not consent. A 2016 attempt by the US EPA to non-voluntarily cancel uses of chlorpyrifos on food crops was ultimately reversed when an industry-friendly administration took control of the agency before the ban was enacted, reinforcing the difficulty that this agency has in cancelling pesticides without the consent of the regulated industry [70].

Of the 13 pesticides identified in this study that are banned in multiple other nations, a few, like dichlobenil and norflurazon, are easy candidates for voluntary cancellation because their use has dropped so much in recent years that continued registration in the USA is increasingly losing cost effectiveness. However the majority are highly used and/or increasing, making a voluntary cancellation less likely. While the non-voluntary cancellation process can be lengthy and tense at times, the US EPA has shown that it can flex its regulatory muscles and ban harmful pesticides without the blessing of the pesticide industry. However, FIFRA gives the US EPA significant discretion on what pesticides it ultimately decides to cancel; for example FIFRA requires a cost-benefit analysis for all harms except those that come from aggregate exposures to humans through food. Because the costs of things like reduced pollination services, reduced water quality, environmental degradation, reduced quality of life and the benefits of maintaining a rich array of biodiversity are extremely difficult to accurately quantify, this cost-benefit analysis largely becomes a qualitative exercise with a high degree of subjectivity and potential for influence by the agrochemical industry.

The goal of this study was to identify the pesticides that different regulatory systems have deemed too harmful for use and compare between nations. It did not seek to compare the effectiveness or robustness of pesticide regulations as a whole between nations. As such, the conclusions here can’t necessarily be generalized to other aspects of pesticide regulation, such as safeguards that do not involve the total banning of a pesticide, the implementation and enforcement of regulations, and regulation compliance.

While a pesticide ban is the most effective method of preventing exposure to a single pesticide, one potential undesirable effect is that it could result in the substitution of another pesticide that has a similar potential for harm [71]. For instance, a ban on one OP pesticide could trigger the greater use of a different pesticide in the same class, resulting in similar risks to humans and many other animals. Alternatively, while the substitution of a banned OP pesticide with a neonicotinoid may lower the risk of harm to humans, it may result in a much higher risk of harm to pollinators due to the higher exposure potential through contaminated pollen and nectar. Therefore, bans can come with tradeoffs and it’s unclear to what extent pesticide bans in these nations have resulted in regrettable substitutions that end up accomplishing little or trade one detrimental risk for another.

Removing a pesticide from use, either voluntarily or non-voluntarily, could have the consequence of disrupting the management of pesticide resistance. Losing a single pesticide may impact the practice of rotating pesticides with different mechanisms of action to delay resistance development. However, if other, safer recommended resistance management steps are taken – such as the halting of prophylactic pesticide use, using non-chemical pest management, scouting for lack of efficacy and practicing smart crop rotation – the overall impact will likely be minor.

It is possible that a pesticide ban or commitment to phase out a pesticide in China or Brazil could be reversed. For example, the newly elected presidential administration in Brazil has been openly hostile to environmental regulations and will likely try to reverse pesticide safeguards in the country in the future [72]. Furthermore, pesticide registrants always have the option to apply for approval of a pesticide that is not currently approved in the EU or USA. Therefore, this list of banned and approved pesticides is a snapshot and subject to change.

What actually constitutes a “ban” is open to interpretation. China and Brazil put in place pesticide bans that, in theory, prohibit their use in the country indefinitely. For the EU and USA, this study considered a pesticide as “banned” if the regulating agency made a unilateral, non-voluntary decision to cancel a pesticide or not approve its use. Some of the pesticides defined as “banned” in the USA and EU were due to failure of the pesticide registrants to pay necessary fees or submit required studies, resulting in non-voluntary cancellation. In these cases, it was impossible to tell whether the studies were not formally submitted due to harmful effects being found that would preclude approval or whether it was an economic decision on the part of the pesticide registrant to not conduct the study or pay fees. Therefore, some pesticides that were designated as “banned” in the USA or EU in this study might more appropriately be designated as “not approved;” however without more information, further refinement was not possible. In addition, voluntary cancellation is not always “voluntary,” and the underlying decisions of most voluntary cancellations are not public information. Regulating agencies can negotiate a voluntary cancellation with pesticide registrants or an impending regulatory action can result in a registrant preemptively cancelling a pesticide voluntarily. So some voluntary cancellations might more appropriately be labeled as “banned” instead of “not approved,” however a lack of publicly available information precluded further refinement.

While it’s not surprising that the EU has banned many pesticides that are still used in the USA, the extent to which this has occurred is quite remarkable. In 2016 the USA used more than 320 million pounds of pesticides that were banned in the EU, accounting for more than a quarter of all agricultural pesticide use (Table 1 and Additional file 5: Table S131). Europe is often criticized by pesticide makers and agricultural interests as being overly protective with burdensome regulations. While the EU has less land dedicated to agriculture than China, its export value of agricultural products is higher than the USA, China and Brazil combined [1]. Therefore, the EU remains highly competitive as a major agricultural power despite having banned many widely-used, potentially hazardous agricultural pesticides.

Of the 25 most commonly used pesticides in the USA, ten – including chloropicrin and paraquat – are banned in at least one of these three agricultural nations [64]. Paraquat and phorate are the only two pesticides still used in the USA that are banned or being phased out in the EU, China and Brazil. Both have been recommended for regulation under the Rotterdam Convention, indicating a growing international concern over their safety [73]. While this treaty does not mandate the banning of listed hazardous chemicals, it provides a mechanism by which countries can essentially “opt-out” from receiving them through trade [74]. Many hazardous chemicals listed in the Rotterdam Convention end up getting banned by countries party to the treaty due to human and environmental health concerns. The USA is just one of six countries in the world that has not ratified the Rotterdam Convention treaty [75].

## Conclusions

Total pesticide bans remain the most effective way to prevent intentional or accidental exposure to highly hazardous pesticides and can catalyze the transition to safer alternatives [76, 77]. Surprisingly, the USA is lagging when it comes to banning or phasing out pesticides that the top agricultural powers have identified as too harmful for use.

This is likely due to deficiencies in pesticide legislation in the USA. FIFRA gives the US EPA significant discretion on which pesticides it ultimately decides to cancel and makes the US EPA-initiated, non-voluntary cancellation process particularly onerous and politically fraught. This, in part, has led to an almost exclusive reliance on industry-initiated, voluntary cancellation of pesticides in the USA.

Without a change in the US EPA’s current reliance on voluntary mechanisms for pesticide cancellations, the USA will likely lag behind its peers in banning these harmful pesticides. Recent mitigation measures finalized for paraquat by the US EPA, which include warning labels, extra training requirements and safer packaging standards that are fully supported by the pesticide industry, indicate that voluntary mitigations will likely be used in lieu of cancellations for at least some of these dangerous pesticides in the future [78].

## Additional files

Additional file 1: Supplemental methods. (PDF 466 kb)

Additional file 2: Sources used to compile the final list of agricultural pesticides. (XLSX 39 kb)

Additional file 3: Approval status of agricultural pesticides in the USA, EU, China and Brazil. (XLSX 133 kb)

Additional file 4: Total pesticide use in the USA in 2016 itemized by active ingredient. (XLSX 14860 kb)

Additional file 5: Total use of pesticides in the USA in 2016 that are banned, being phased out or not approved in other agricultural nations. (XLSX 35 kb)

Additional file 6: Trends in use of pesticides in the USA that are banned in at least two of three other agricultural nations (2007–2016). (PDF 578 kb)

## Abbreviations

ANVISA: Agência Nacional de Vigilância Sanitária of Brazil
CDC: Centers for Disease Control and Prevention
EU: European Union
FFDCA: Federal Food, Drug, and Cosmetic Act
FIFRA: Federal Insecticide, Fungicide, and Rodenticide Act
FQPA: Food Quality Protection Act
MOA: Ministry of Agriculture
MOE: Ministry of the Environment
OP: Organophosphate Pesticide
PAN: Pesticide Action Network
US EPA: United States Environmental Protection Agency
USA: United States of America
USGS: United States Geological Survey
WHO: World Health Organization
