# Supplementary material for: The USA lags behind other agricultural nations in banning harmful pesticides
Source: Environ Health. 2019 Jun 7;18:44. doi: 10.1186/s12940-019-0488-0 (PMC6555703; doi:10.1186/s12940-019-0488-0)
Supplement: Supplementary file 1 — Supplemental methods. (PDF 466 kb) [file 12940_2019_488_MOESM1_ESM.pdf]

Supplemental Methods

<u>Pesticide List</u>

I used multiple sources to compile a comprehensive list of pesticides that have been used in agriculture in the USA since 1970, when the US EPA was formed. The United States Geological Survey (USGS) has collected data on pesticide use specifically in agriculture and maintains a list of pesticides that have been used since 1992 online [1]. Because the regulatory status of *biological* pesticides would be very difficult to compare between countries, those pesticides were removed from the list – leaving all *chemical* pesticides that have been used in USA agriculture from 1992-2016 (the most recent year data were available). In order to identify pesticides that may have been used in USA agriculture but banned or cancelled before 1992, I compiled Annex III pesticides of the Rotterdam Convention and Annex A and B pesticides of the Stockholm Convention [2, 3]. In addition, I compiled pesticides from a 1990 US EPA list of suspended, cancelled and restricted pesticides [4]. Pesticides from those lists that were non-agricultural, or whose status in all three nations (EU, China and Brazil) could not be determined, were removed before adding to the final list. The final 508 pesticides in the list, as well as information about list sources and reasons for removal, are outlined in Additional File 2.

<u>Data Sources for Pesticide Status</u>

The approval status of each pesticide in the USA, EU, China and Brazil was identified through various sources. The European Commission and ANVISA maintain easy-to-use resources that display pesticide status in the EU and Brazil, respectively – making information transparent and easily accessible. On the other hand, identifying pesticide status in the USA was time consuming and difficult with public information decentralized in various US EPA databases, documents and Federal Register notices. There was no resource to identify pesticide status in China short of searching through thousands of MOA announcements, so pesticide status was largely identified from a 2015 report published by the Food and Agriculture Organization of the United Nations.

The following information and references can be found in Additional File 3: Tables S6-S9:

> **Brazil**: Pesticide status in Brazil was largely identified from lists maintained by the Agência Nacional de Vigilância Sanitária of Brazil (ANVISA), which is the Brazilian Health Regulatory Agency, and the Rotterdam Convention list of banned pesticides. For pesticides that are being phased out and not yet banned, ANVISA resolutions were used as the information source. Google translate was used to convert Portuguese to English for review of ANVISA documents.
>
> **China**: Information on pesticide status in China was largely identified from an extensive 2015 study from the Food and Agriculture Organization of the United Nations on pesticide regulation in Asia and the Rotterdam Convention list of banned pesticides. Every effort was made to reference a specific announced ban from the Ministry of Agriculture (MOA) of the

People's Republic of China – however this was not possible in every case. The MOA recently announced planned phase outs of multiple pesticides and 3 independent media reports were used as confirmation of this commitment. While information on *pesticide bans and phase outs* in China is current in this analysis, pesticide *approval* status is only current as of 2015. Google translate was used to convert Mandarin to English for review of Chinese MOA documents.

European Union: Pesticide status in the EU was identified from a database maintained by the European Commission (EC) and the Rotterdam Convention list of banned pesticides. For most pesticides that have been "banned" or "not approved," there is reference to a European Commission Regulation/Decision or Council of the European Union Decision/Directive. These cited regulations, decisions and directives were analyzed to determine whether the pesticide fit the "banned" or "not approved" definitions outlined in the Methods section. If no regulation, decision or directive was provided in the EC database, that pesticide was designated "not approved."

United States of America: Pesticide status in the USA was identified from the US EPA's Pesticide Product and Label System (PPLS) database and a 1990 report from the US EPA on suspended, cancelled and restricted pesticides. For pesticides that had no active label for use on agricultural crops, two additional sources were utilized. The US EPA's Pesticide Chemical Search Tool and the Federal Register – which is an archived accounting of regulatory actions taken by the US government – were searched for details on how and why the pesticide was cancelled. Each federal register notice was analyzed to determine whether the pesticide fit the "banned" or "not approved" definitions outlined above.

1. US Geological Survey. USGS NAWQA: The Pesticide National Synthesis Project. Estimated Annual Agricultural Pesticide Use. Pesticide Use Maps. https://water.usgs.gov/nawqa/pnsp/usage/maps/compound_listing.php. Accessed 19 Jun 2018.

2. Rotterdam Convention on the Prior Informed Consent Procedure for Certain Hazardous Chemicals and Pesticides in International Trade. Annex III Chemicals. http://www.pic.int/TheConvention/Chemicals/AnnexIIIChemicals/tabid/1132/language/en-US/Default.aspx. Accessed 16 Jun 2018.

3. Stockholm Convention on Persistant Organic Pollutants. All POPs listed in the Stockholm Convention. http://chm.pops.int/TheConvention/ThePOPs/AllPOPs/tabid/2509/Default.aspx. Accessed 19 Jun 2018.

4. US Environmental Protection Agency. Suspended, Cancelled, and Restricted Pesticides. Washington, D.C.; 1990. https://nepis.epa.gov/Exe/ZyPDF.cgi/20011E0G.PDF?Dockey=20011E0G.PDF.
