# Supplementary material for: The USA lags behind other agricultural nations in banning harmful pesticides
Source: Environ Health. 2019 Jun 7;18:44. doi: 10.1186/s12940-019-0488-0 (PMC6555703; doi:10.1186/s12940-019-0488-0)
Supplement: Supplementary file 6 — Trends in use of pesticides in the USA that are banned in at least two of three other agricultural nations (2007–2016). (PDF 578 kb) [file 12940_2019_488_MOESM6_ESM.pdf]

a

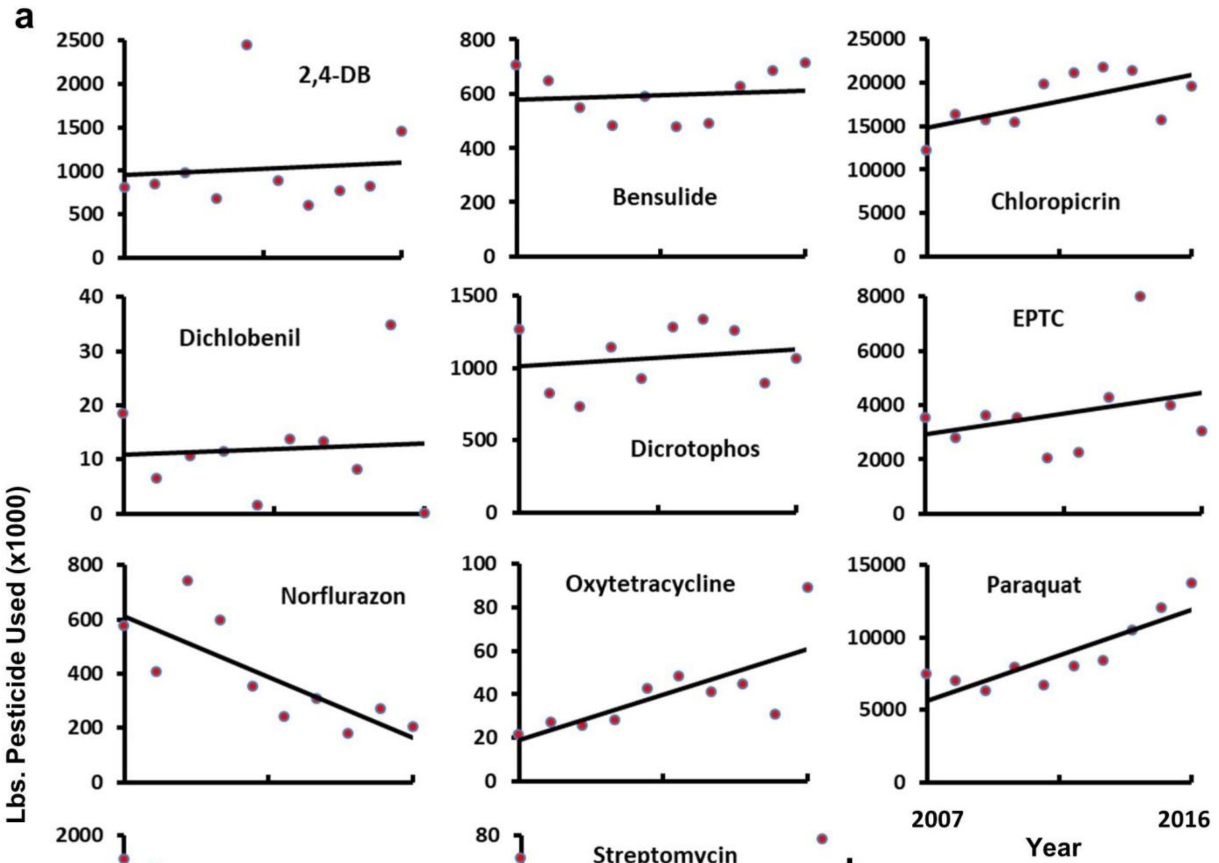

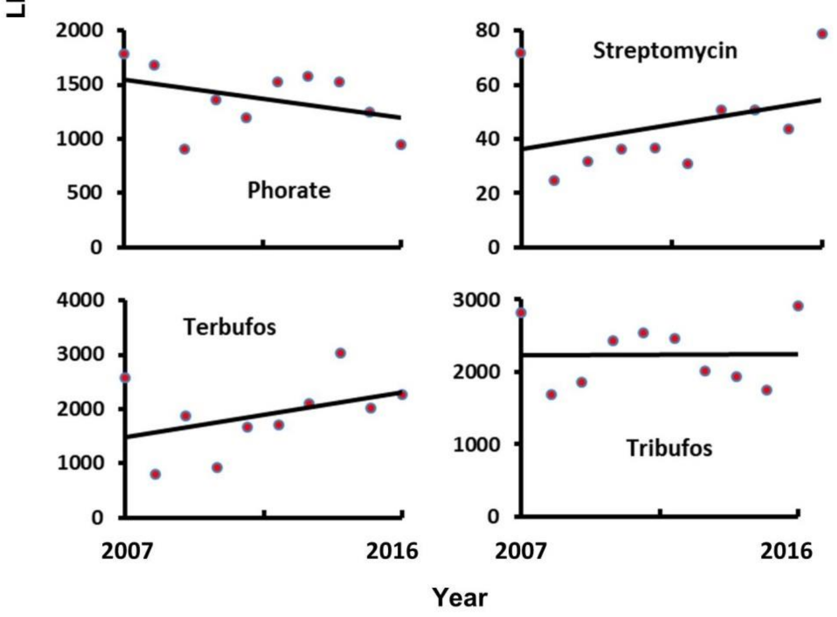

b

Pesticide	F	*p*
**2,4-DB***	0.11	0.754
**Bensulide**	0.12	0.739
**Chloropicrin**	5.12	0.053
**Dichlobenil**	0.04	0.845
**Dicrotophos**	0.30	0.598
**EPTC***	0.68	0.434
**Norflurazon**	12.70	**0.007**
**Oxytetracycline**	8.92	**0.017**
**Paraquat**	22.48	**0.001**
**Phorate**	1.49	0.257
**Streptomycin***	1.49	0.257
**Terbufos**	1.59	0.243
**Tribufos**	0.00	0.968

* Log transformed

Supplemental Figure 1. Trends in Use of Pesticides in the USA that are Banned in at Least Two of Three Other Agricultural Nations. **a)** Total pesticide use in the USA in pounds (lbs.) was plotted for each year between 2007 and 2016 for each of 13 pesticides that have been banned or are being phased out in at least two of the following places: European Union, China and Brazil. Each graph contains a linear trend line. **b)** Results of linear regression analyses that were conducted for each pesticide over the 10-year

period. Data were log-transformed where indicated and the degrees of freedom (df) for each pesticide dataset equals 9. Bold p-values were statistically significant ($p<0.05$). Pesticides highlighted in yellow had a significant downward trend, pesticides highlighted in blue had a significant upward trend and those that were not highlighted had no significant change over time.
